# Ferromagnetic Interlayer Exchange Coupling in Magnetic Topological Insulator Sandwich Heterostructures

**DOI:** 10.1002/advs.202514562

**Published:** 2026-01-27

**Authors:** Enayet Hossain, Grace L. Causer, Qile Li, Sergey Rubanov, Kaijian Xing, James Blyth, Mohammad T. H. Bhuiyan, Mengting Zhao, Matthew Gebert, Michael S. Fuhrer, Mark T. Edmonds

**Affiliations:** ^1^ School of Physics and Astronomy Monash University Clayton Victoria Australia; ^2^ ARC Centre of Excellence for Future Low‐Energy Electronics Technologies (FLEET) Monash University Clayton Victoria Australia; ^3^ Department of Physics University of Dhaka Dhaka Bangladesh; ^4^ Ian Holmes Imaging Centre (IHIC), Bio21 Institute University of Melbourne Melbourne Victoria Australia

**Keywords:** anomalous Hall effect, antiferromagnetism, interlayer exchange coupling, magnetic topological insulator, proximity magnetization

## Abstract

A single septuple layer (SL) of MnBi_2_Te_4_ is a promising 2D ferromagnetic insulator for integrating magnetism with topology in van der Waals heterostructures, using topological insulators such as the nearly lattice matched Bi_2_Te_3_ with quintuple‐layer (QL) units. Here, electrical transport measurements are performed on 1 SL MnBi_2_Te_4_/*n* QL Bi_2_Te_3_/1 SL MnBi_2_Te_4_ sandwich heterostructures (*n* = 0–4) to investigate the role of Bi_2_Te_3_ spacer thickness in tuning interlayer magnetic interactions. Magnetotransport reveals that even 1 QL Bi_2_Te_3_ is sufficient to switch the intrinsic antiferromagnetic coupling in 2 SL MnBi_2_Te_4_ to ferromagnetic, evidenced by Hall hysteresis and the absence of spin‐flop transitions. Increasing *n* leads to a monotonic decrease in coercivity and Curie temperature, reflecting progressively weaker interlayer coupling, with a simultaneous enhancement in anomalous Hall response at *n* = 4. These results demonstrate reversible control of spin configuration by magnetic field and confirm the role of magnetic proximity‐induced exchange coupling in determining the interlayer magnetic ground state, highlighting this atomic‐scale spacer‐engineered heterostructure as a compelling platform for spintronic applications and tunable symmetry‐broken topological quantum phases.

## Graphical abstract

A thin topological insulator (Bi_2_Te_3_) inserted between two single magnetic septuple layers of MnBi_2_Te_4_ is shown to convert the intrinsic antiferromagnetic interlayer coupling into robust ferromagnetism. As the Bi_2_Te_3_ spacer thickness increases from 1 to 4 quintuple layers, the weakening coercivity, Curie temperature, and interlayer coupling reveal tuneable magnetic interactions and offer a pathway toward higher‐temperature quantum anomalous Hall states.

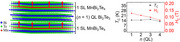

## 1 Introduction

As an intrinsic magnetic topological insulator (MTI), MnBi_2_Te_4_ has emerged as a model system for exploring the interplay between magnetism and topology [1, 2, 3, 4, 5, 6, 7]. MnBi_2_Te_4_ hosts gapped Dirac surface states arising from spontaneous time‐reversal symmetry breaking, achieved without the need for extrinsic magnetic doping [2, 3, 7, 8, 9, 10, 11, 12]. This makes MnBi_2_Te_4_ a promising platform for realizing novel quantum phases, including the quantum anomalous Hall effect (QAHE) [2, 9, 10, 13, 14] and axion insulator states [15], offering a robust alternative to magnetically doped topological insulators, which often suffer from disorder and limited thermal stability [1, 4, 6, 8, 16, 17, 18].

**FIGURE 1:**
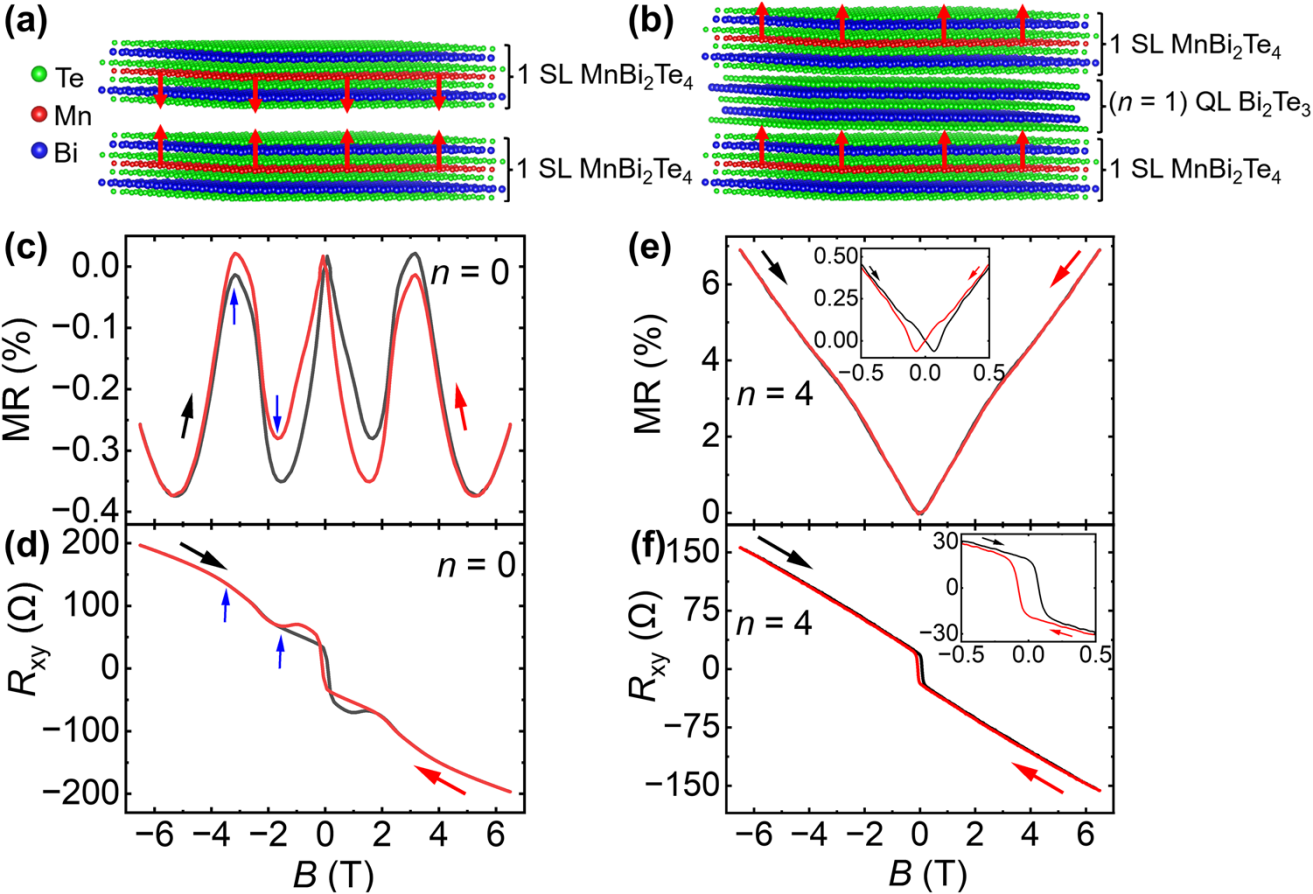
Transition in magnetic behaviour of 1 SL MnBi_2_Te_4_/*n* QL Bi_2_Te_3_/1 SL MnBi_2_Te_4_ heterostructure from AFM interlayer order at *n* = 0 (corresponding to 2 SL MnBi_2_Te_4_) to FM interlayer order at *n* > 0. (a,b) Atomically resolved structural models showing the layer‐by‐layer stacking along the c‐axis. (a) 2 SL MnBi_2_Te_4_ (*n* = 0) with interlayer AFM spin alignment indicated by red arrows. (b) 1 SL MnBi_2_Te_4_/1 QL Bi_2_Te_3_/1 SL MnBi_2_Te_4_ heterostructure, where the Bi_2_Te_3_ spacer modifies the magnetic coupling between the Mn layers, leading to FM interlayer order. (c) Magnetoresistance (MR) and (d) Hall resistance (*R*_xy_) for 2 SL MnBi_2_Te_4_ measured at 3 K. The emergence of a spin‐flop transition is evident, with blue arrows indicating the critical fields associated with spin reorientation. (e) MR and (f) *R*_xy_ of a 1 SL MnBi_2_Te_4_/4 QL Bi_2_Te_3_/1 SL MnBi_2_Te_4_ heterostructure at 3 K, showing clear FM behavior. Black and red arrows indicate increasing and decreasing field directions, respectively. All *R*_xy_ data were anti‐symmetrized to eliminate *R*_xx_ contributions.

A key advantage of MnBi_2_Te_4_ lies in its layered van der Waals structure, where each septuple layer (SL) consists of a Te‐Bi‐Te‐Mn‐Te‐Bi‐Te stacking sequence along the crystallographic c‐axis [1, 3, 4, 6, 19, 20, 21]. Within each SL, the Mn magnetic moments align ferromagnetically, making 1 SL MnBi_2_Te_4_ a 2D ferromagnet. In addition, the absence of topological surface states renders 1 SL MnBi_2_Te_4_ a large gap conventional insulator [22]. However, interlayer exchange coupling between adjacent SLs is antiferromagnetic (AFM), resulting in an overall A‐type AFM order for multilayer MnBi_2_Te_4_ [4, 19] as shown by the red arrows in Figure 1a. This interlayer coupling causes a spin‐flop transition under a large external magnetic field applied parallel to the AFM moments, posing a challenge for achieving robust out‐of‐plane ferromagnetic (FM) order required for high‐temperature QAHE [23]. Despite its potential, ultra‐thin MnBi_2_Te_4_ suffers from magnetic disorder that causes a breakdown of topological protection and suppression of the QAHE temperature [17, 23].

To overcome magnetic disorder, inducing magnetism in a topological insulator via magnetic proximity coupling is a promising alternative [1, 2, 8, 16]. In this scheme, a non‐magnetic topological insulator such as Bi_2_Te_3_, following the quintuple layer (QL) stacking order Te‐Bi‐Te‐Bi‐Te and separated by weak van der Waals forces [8, 21, 24], is sandwiched between two 2D FM insulators, enabling magnetization without direct doping, but via interfacial exchange interactions [8, 25, 26, 27, 28, 29]. Figure 1b illustrates this concept: where a Bi_2_Te_3_ spacer is inserted between two 1 SL MnBi_2_Te_4_ layers in order to modify the interlayer magnetic coupling, switching it from AFM‐to‐FM through interfacial proximity‐induced interactions [29, 30].

Numerous studies have focused on engineering magnetic TI heterostructures to realize topological phases. Early demonstrations of the QAHE in thin films of Cr‐doped (Bi, Sb)_2_Te_3_ [10] established the feasibility of magnetic topological phases, but disorder inherent in such doped systems significantly suppressed the quantization temperature. Jiang et al. demonstrated a 3‐5‐3 MTI heterostructure, where an undoped 5 QL (Bi, Sb)_2_Te_3_ is sandwiched between two Cr‐doped 3 QL (Bi, Sb)_2_Te_3_, enabling the concurrence of QAHE and topological Hall effect (THE) [31]. However, due to the disorder in magnetically doped TIs, attention shifted toward intrinsic MTIs and proximity‐coupled heterostructures. For example, Sitnicka et al. investigated MnBi_2_Te_4_/(Bi_2_Te_3_)_n_ superlattices, highlighting how structural disorder modifies magnetic interactions and surface electronic states critical to topological transport [32]. Otrokov et al. proposed that placing a TI such as Bi_2_Te_3_ adjacent to MnBi_2_Te_4_ could induce magnetic proximity, opening Dirac surface gaps and enabling QAHE at elevated temperatures [33]. Later, this concept was experimentally supported by Li et al., who reported a sizeable (∼75 meV) magnetic gap in a ferromagnet–TI–ferromagnet heterostructure, predicting its potential for higher temperature QAHE [25]. More recently, Yao et al. investigated the mechanisms by which Bi_2_Te_3_/MnBi_2_Te_4_ ratio and Mn–Mn interlayer distance influences the coexistence of AFM and FM orders in MnBi_2_Te_4_/Bi_2_Te_3_ heterostructures, composed of alternating SLs of MnBi_2_Te_4_ and QLs of Bi_2_Te_3_ [34].

However, these previous structures often involve repeated superlattices, non‐ MnBi_2_Te_4_ based magnetic layers, or lack clear symmetry and thickness control, making it difficult to isolate the intrinsic role of magnetic proximity in magnetic insulator–topological insulator–magnetic insulator interfaces. Therefore, unlike magnetically doped layers or complex superlattices, in this study, we realize and investigate a sandwich heterostructure–1 SL MnBi_2_Te_4_/*n* QL Bi_2_Te_3_/1 SL MnBi_2_Te_4_–which offers atomic‐level control over thickness and interface quality, enabling a clean platform to isolate proximity‐induced magnetic effects. By systematically varying the Bi_2_Te_3_ spacer thickness (*n* = 0 to 4), we directly tune the interlayer coupling and uncover a transition from AFM‐to‐FM order, alongside enhanced anomalous Hall response. In addition, our novel use of monolayer MnBi_2_Te_4_ as a wide‐gap magnetic insulator for proximity‐induced magnetism, rather than the topological electronic layer itself, avoids the problems of disorder which plague MnBi_2_Te_4_, and paves the way for low disorder magnetic topological devices based on high‐quality MBE‐grown Bi_2_Te_3_‐family materials. This minimal and precisely engineered structure demonstrates a promising route for exploring tunable magnetic topological quantum phases.

## 2 Results and Discussion

High quality 1 SL MnBi_2_Te_4_/*n* QL Bi_2_Te_3_/1 SL MnBi_2_Te_4_ heterostructures (where *n* = 0 to 4) were grown via molecular beam epitaxy (MBE) on strontium titanate (SrTiO_3_), STO (111) substrates. Beyond the structural information obtained from reflection high‐energy electron diffraction (RHEED) and X‐ray diffraction (XRD), cross‐sectional transmission electron microscopy (TEM) and electron energy loss spectroscopy (EELS) reveal atomically sharp interfaces and show that Mn is present only in the MnBi_2_Te_4_ layers and completely absent in the Bi_2_Te_3_ spacer, thereby confirming the chemically sharp and intended layer sequence of the heterostructure (see methods and supporting information for the detailed growth procedure and structural characterization including XRD, TEM and EELS).

To explore the influence of interlayer coupling on magnetotransport behavior, we began by studying a 2 SL MnBi_2_Te_4_ film without any non‐magnetic Bi_2_Te_3_ spacer. Figure 1c,d displays the magnetoresistance (MR = $\frac{R_{\mathrm{xx}}\left(B\right)-R_{\mathrm{xx}}\left(B=0\right)}{R_{\mathrm{xx}}\left(B=0\right)}$) and Hall resistance (*R*_xy_) of the 2 SL MnBi_2_Te_4_ film (*n* = 0) at 3 K, showing clear features of spin‐flop transition arising from intrinsic AFM interlayer coupling between neighboring SLs. The magnetotransport response of the 2 SL MnBi_2_Te_4_ film reflects a continuous evolution of magnetic phases under an external field, consistent with prior reports [19, 20]. As shown in Figure 1c,d, the MR and Hall signals track the same sequence of field‐driven spin configurations. At near zero fields (*B* < 0.071 T), the system remains in its intrinsic AFM state, with small spin reorientations causing a slight increase in MR. At *B* ≈ 0.071 T, the onset of the spin‐flop transition occurs as the external field starts to overcome the interlayer AFM coupling. The adjacent SL spins tilt away from their antiparallel alignment, reducing spin scattering and resulting in a decrease in MR. During this state, R_xy_ exhibits a complex, non‐square hysteresis loop, providing complementary evidence of the spin‐flop transition and reflecting the gradual canting of adjacent SL spins [19].

With increasing fields, the spin continues to cant and a full spin‐flop transition occurs at the critical field *B*_C.sf_ = 1.7 T, where the system enters the canted antiferromagnetic (CAFM) phase [19, 20]. Both transport channels capture this transition: MR shows a slope change, and the Hall curve displays a well‐defined kink at the same field, confirming the spin‐flop transition between AFM and CAFM states. In the CAFM regime, spins progressively align with the field, suppressing spin‐disorder scattering. The subsequent gradual increase in MR arises primarily from the classical orbital (Lorentz‐force) contribution [35], while the non‐linear Hall response reflects the evolving spin canting. MR reaches its maximum near the saturation critical field *B*_C.sat_ = 3.2 T, marking the transition to a FM state with almost fully aligned spins [36]. Beyond 3.2 T, the field further stabilizes the FM order, reducing magnetic scattering until ≈5.2 T. At higher fields (*B*> 5.2 T), MR increases again due to the quadratic orbital magnetoresistance, independent of spin configuration.

Overall, the spin‐flop transition and the associated critical fields observed in both the magnetoresistance and Hall resistance data, are nearly identical, confirming the field‐driven AFM‐to‐FM transition of the interlayer coupling in the 2 SL MnBi_2_Te_4_ film [1, 20, 37, 38, 39]. A schematic illustrating how the complex spin configuration in 2 SL MnBi_2_Te_4_ evolves with external magnetic field is provided in Figure S4(i) to aid interpretation of the MR and Hall responses [19]. To further analyze magnetic switching in 2 SL MnBi_2_Te_4_, the anomalous Hall resistance (*R*_AH_) was extracted from the total *R*_xy_ and quantitatively decomposed into the expected two components *R*_AH1_ and *R*_AH2_ [19, 20] as shown in Figure S4–S6.

To investigate how the interlayer AFM coupling of 2 SL MnBi_2_Te_4_ can be modulated, we fabricated a 1 SL MnBi_2_Te_4_/4 QL Bi_2_Te_3_/1 SL MnBi_2_Te_4_ heterostructure, and the corresponding transport data are shown in Figure 1e,f. Here, the spin‐flop features observed for the pristine 2 SL MnBi_2_Te_4_ film are completely absent for the heterostructure. Instead, the MR curve reveals butterfly‐shaped hysteresis (discussed in detail in Figure 3d–g) near zero field and a monotonic field‐dependent increase with no peak or negative MR features—indicating suppression of interlayer AFM coupling. Concurrently, the *R*_xy_ curve is characterized by a pronounced square‐shaped hysteresis loop with a sharp coercive field, followed by a linear high‐field dependence, indicating uniform FM alignment of magnetic moments without interlayer AFM competition [10, 34]. To provide a clearer view of magnetic hysteresis, the inset of Figure 1e,f zooms into the low‐field region to emphasize the loop‐like features in both MR and *R*_xy_. These observations suggest that inserting the Bi_2_Te_3_ spacer between SLs of MnBi_2_Te_4_ transforms the interlayer exchange interaction from AFM‐to‐FM, likely arising from proximity‐induced interactions across the topological spacer [26, 29, 30].

**FIGURE 2:**
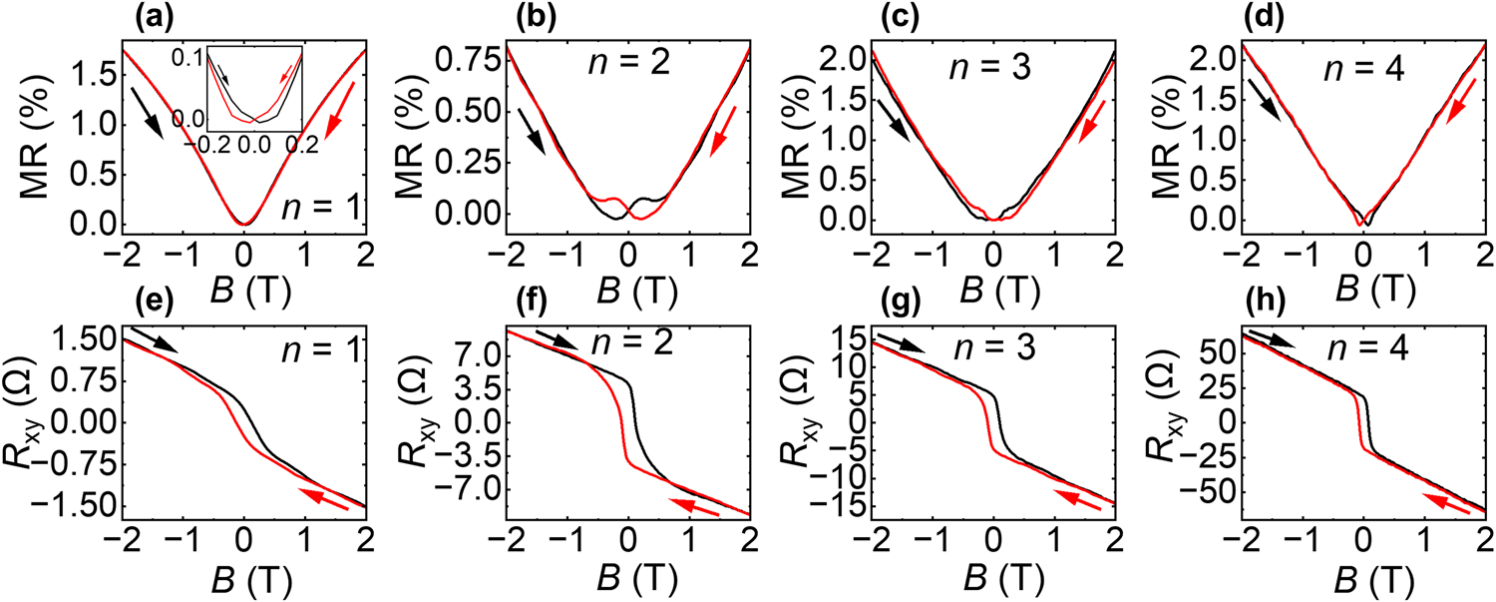
Magnetotransport characterization of 1 SL MnBi_2_Te_4_/*n* QL Bi_2_Te_3_/1 SL MnBi_2_Te_4_ heterostructures with varying Bi_2_Te_3_ thickness (*n* = 1 to 4) measured at 3 K. (a–d) MR curves, showing the low‐field butterfly‐shaped hysteresis. (e–h) Anti‐symmetrized Hall resistance (*R*_xy_) for the same samples, highlighting clear hysteresis for all *n*, indicative of interlayer FM order mediated by Bi_2_Te_3_ spacer. Black and red arrows indicate increasing and decreasing field directions, respectively.

Building on the observation that a 4 QL Bi_2_Te_3_ spacer induces interlayer ferromagnetism, we now explore how this magnetic transition develops with reduced spacer thickness. Figure 2a–d shows the MR and Figure 2e–h shows *R*_xy_, measured at 3 K for heterostructures with *n* = 1–4, enabling a systematic comparison of the interlayer magnetic interactions as the spacer layer thickness is increased.

For *n* = 2–4 in Figure 2b–d, the MR curves exhibit butterfly‐shaped hysteresis, indicative of interlayer FM coupling. For *n* = 1 (Figure 2a), although the main MR curve lacks a pronounced butterfly shape, a closer look at the low‐field region—highlighted in the inset— reveals a weak butterfly hysteresis, suggesting that magnetic switching does occur but the hysteresis is less pronounced at this spacer thickness.

On the other hand, a clear trend emerges in *R*_xy_, shown in Figure 2e–h, shows consistent and well‐defined square‐shaped hysteresis loops across all spacer thicknesses. While all *R*_xy_ is plotted here in the field range of ±2 T to highlight the sharp switching behavior, the complete field dependent *R*_xy_ over the full ± 6.5 T range are provided in Figure S7.

All heterostructures show qualitatively similar behavior, distinct from the spin‐flop signature in the 2 SL MnBi_2_Te_4_ film (*n* = 0 case). The high‐field linear dependence of *R*_xy_ vs. *B* data without any distinct anomaly or kink, suggests that our sandwich heterostructures have no spin‐flop transition even at 3 K, indicating complete interlayer FM ordering with strong perpendicular magnetic anisotropy (PMA) [40, 41, 42], rather than AFM or uncompensated AFM ordering which occurs for even/odd MnBi_2_Te_4_ layers respectively of 2 SL thickness or greater [17, 19, 20].

The consistent interlayer FM behavior across different topological insulator spacers demonstrates that even a single QL of Bi_2_Te_3_ is sufficient to decouple adjacent MnBi_2_Te_4_ SLs, transforming the magnetic ground state from AFM‐to‐FM. While the hysteresis loops show slight variations with increasing *n* (discussed in detail below), the overall switching behavior is robust, highlighting the effectiveness and tunability of the heterostructure with varying the Bi_2_Te_3_ QL thickness.

**FIGURE 3:**
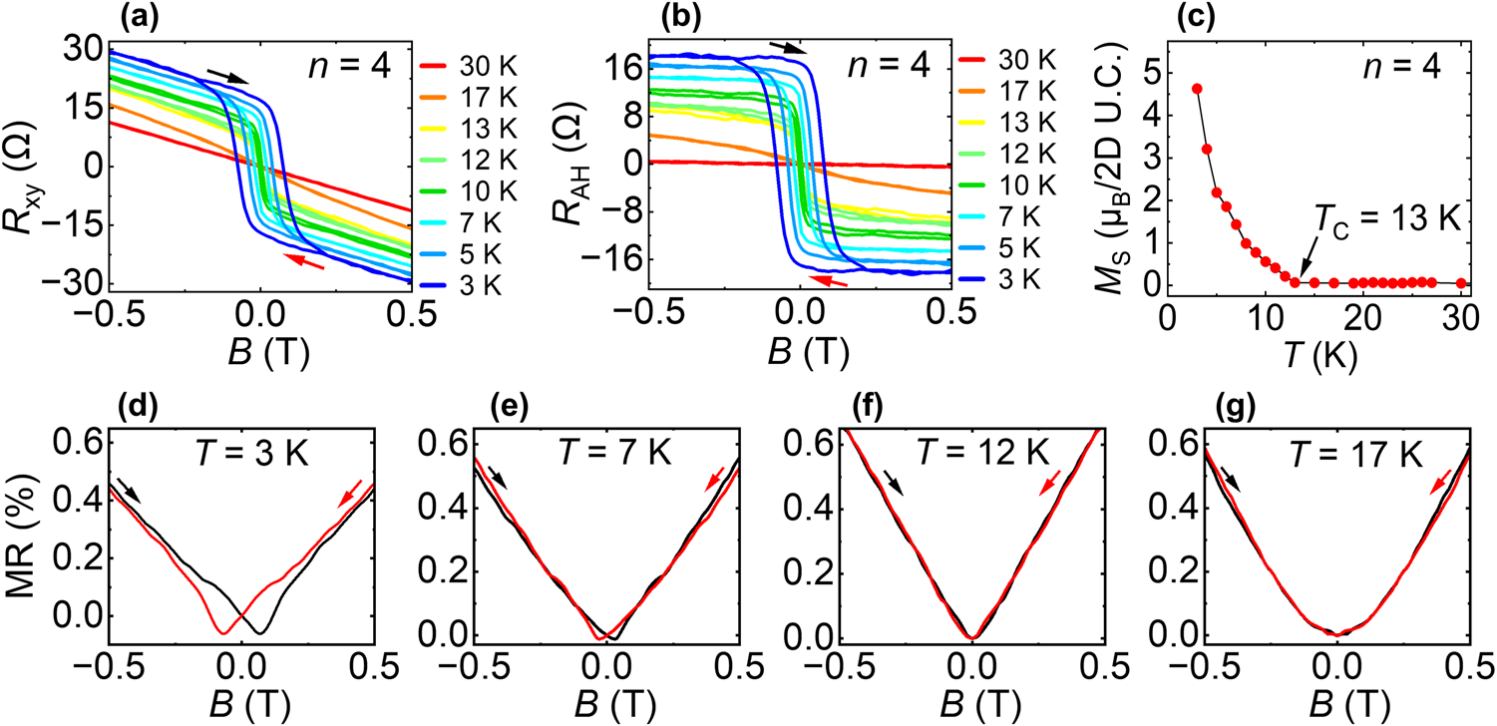
Temperature‐dependent transport and magnetometry of 1 SL MnBi_2_Te_4_/4 QL Bi_2_Te_3_/1 SL MnBi_2_Te_4_ heterostructure. (a) Magnetic field dependence of Hall resistance (*R*_xy_) measured at different temperatures, showing the evolution of hysteresis. (b) Anomalous Hall resistance (*R*_AH_) curves determined by subtracting the ordinary Hall background from the total *R*_xy_. (c) Temperature dependent saturation magnetization (*M*_S_), confirming the *T*_C_ of the heterostructure. (d–g) Magnetoresistance (MR) measured at 3, 7, 12, and 17 K. Black and red arrows represent increasing and decreasing field directions, respectively.

To further explore the robustness of the FM state, we conducted temperature‐dependent transport measurements on 1 SL MnBi_2_Te_4_/4 QL Bi_2_Te_3_/1 SL MnBi_2_Te_4_ heterostructure. This sample serves as a representative for probing how FM signatures respond to increasing temperature. As shown in Figure 3, we focus on the low‐field regime of both MR and *R*_xy_ to capture and understand the magnetic switching and anomalous Hall signatures. The Hall data for heterostructures with thinner Bi_2_Te_3_ spacers (*n* = 1–3) are included in Figure S8.

The *R*_xy_ for *n* = 4 heterostructure, measured at low temperature, from 3 to 12 K, shows a distinct hysteresis loop indicating interlayer FM order [43]. As shown in Figure 3a, the 3 K trace (blue) shows a loop extending out to nearly 0.1 T, corresponding to the butterfly‐shaped MR hysteresis observed at the same temperature (discussed below). The *R*_xy_ hysteresis then gradually weakens with increasing temperature and disappears entirely at 13 K (yellow trace).

To isolate the anomalous Hall resistance, *R*_AH_, a linear background subtraction was performed on the *R*_xy_ data associated with the ordinary Hall signal, *R*_OH_ (i.e. *R*_xy_ = *R*_OH_ + *R*_AH_), and the resulting *R*_AH_ curves are shown in Figure 3b. The *R*_AH_ curves follow the same temperature‐dependent trend, with the hysteresis vanishing at and above 13 K. We call this the Curie temperature (*T*_C_)—marking the transition from a FM phase to a paramagnetic state, above which thermal fluctuations dominate and the magnetic moments become disordered [5].

To corroborate the *T*_C_, we performed SQUID magnetometry on the 1 SL MnBi_2_Te_4_/4 QL Bi_2_Te_3_/1 SL MnBi_2_Te_4_ heterostructure. As shown in Figure 3c, the saturation magnetization (*M*_S_) at 3 K is 4.63 µ_B_/2D unit cell and decreases steadily with temperature, reaching zero at around 13 K. This excellent agreement between transport and magnetometry data confirms the *T*_C_ of the *n* = 4 heterostructure. In addition, the magnetization observed in our heterostructure is comparable to that of the bulk MnBi_2_Te_4_ (4.08 µ_B_/Mn) [44].

Figure 3d–g investigates the temperature dependence of the MR of *n* = 4 heterostructure [the MR at 3 K is shown in the inset of Figure 1e]. From 3 to 7 K, MR curves are characterized by butterfly‐shaped hysteresis with two distinct dips at near zero field. In magnetic TIs, strong FM ordering at low temperature forms finite domains with PMA [40], creating gapless chiral domain‐wall bound states during magnetization switching near zero field—giving rise to the butterfly feature [30, 45]. Then at higher temperatures (e.g., 12 K in Figure 3f), thermal energy disrupts domain alignments, weakening PMA and rotating the easy axis from out‐of‐plane to in‐plane. This reduces the energy barrier for magnetization reversal, eliminating the butterfly shape in MR [46].

**FIGURE 4:**
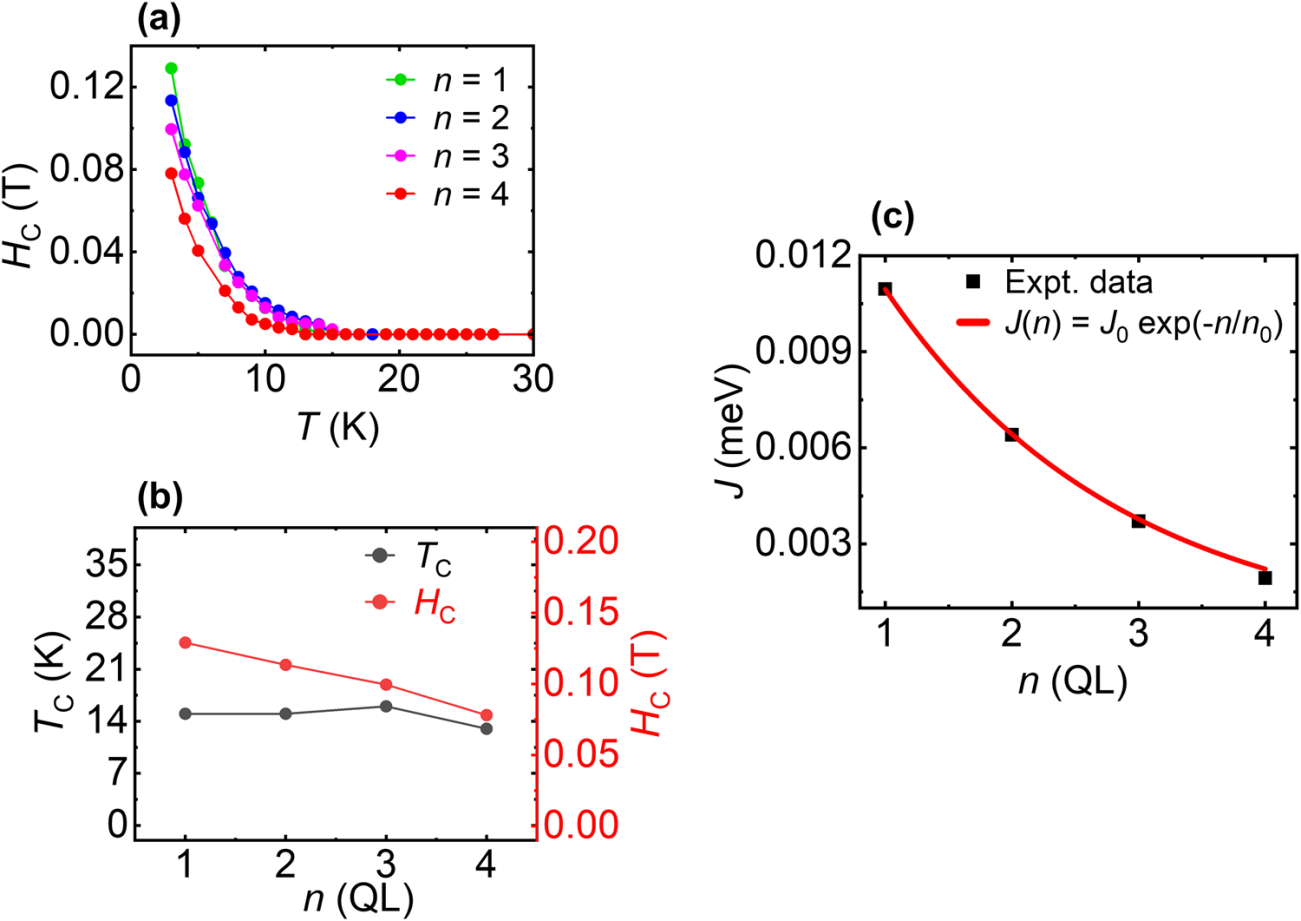
Dependence of magnetic properties on Bi_2_Te_3_ spacer thickness in 1 SL MnBi_2_Te_4_/*n* QL Bi_2_Te_3_/1 SL MnBi_2_Te_4_ heterostructure. (a) Temperature dependence of coercive field (*H*_C_) extracted from *R*_xy_ vs *B* curves. (b) Curie temperature (*T*_C_) and coercive field (*H*_C_) at 3 K as a function of *n* Bi_2_Te_3_ QLs. (c) Interlayer magnetic coupling strength (*J*) for *n* = 1–4 at 3 K.

We now examine how the coercivity (*H*_C_) and *T*_C_ changes with *n*. Figure 4a presents the temperature dependence of *H*_C_ extracted from *R*_xy_ loops for all four heterostructures (*n* = 1 to 4) between 3 and 30 K. The *H*_C_ was determined from the magnetic field where the hysteresis loop crosses zero *R*_xy_, corresponding to the magnetization reversal. The *H*_C_ values from increasing and decreasing field sweeps are identical, indicating symmetric magnetic switching without exchange bias. Note that the *H*_C_ vs *T* data for the 2 SL MnBi_2_Te_4_ structure (*n* = 0) are excluded from Figure 4a, since its intrinsic AFM interlayer coupling leads to a different spin‐flop mechanism that does not follow the monotonic behaviour observed in the heterostructures. For *n* = 1, the *H*_C_ is 0.13 T at 3 K and exhibits a systematic decrease with increasing temperature, ultimately vanishing at 15 K—the *T*_C_. The other heterostructures follow the similar trend: *H*_C_ decreases monotonically and disappears near their respective *T*_C_ values.

At 3 K, as shown in Figure 4b, *H*_C_ varies slightly among the samples, with values of 0.13 T (*n* = 1), 0.113 T (*n* = 2), 0.10 T (*n* = 3) and 0.078 T (*n* = 4), despite these variations, *T*_C_ values remain relatively consistent across the heterostructures, falling in a narrow range between 15 and 16 K—except for *n* = 4, where *T*_C_ appears slightly reduced to 13 K. The systematic change in *H*_C_ thus serves as a direct indicator of variations in the interlayer magnetic coupling. In addition, because defects can influence the coercivity by acting as nucleation sites for reversed magnetic domains, we further examined the magnetic coupling between the MnBi_2_Te_4_ layers. In Figure 4c, we have quantitatively estimated the interlayer exchange coupling strength (*J*) for the 1 SL MnBi_2_Te_4_/*n* QL Bi_2_Te_3_/1 SL MnBi_2_Te_4_ heterostructures using the formula based on the Stoner–Wohlfarth model [47, 48] (see section VII of SI for details). The extracted *J* values show a clear thickness dependence, decreasing rapidly with increasing Bi_2_Te_3_ spacer thickness, indicative of a finite‐range interlayer coupling.

To further understand this behaviour, the resulting *J(n)* values were fitted using an exponential decay model *J*(*n*) = *J*_0_ $e^{-n/n_{0}}$, which captures the suppression of interlayer coupling with increasing QL thickness [49, 50, 51]. The exponential fit reproduces the experimental trend well, confirming the expected decay of magnetic coupling strength through the nonmagnetic spacer. From this fit, we obtained *J*_0_ = 0.0187 meV, which represents the interlayer AFM coupling strength of 2 SL MnBi_2_Te_4_ sample. The extracted characteristic decay length, *n*_0_ = 1.88 indicates that the interlayer coupling strength drops to 37% within 1.88 QL of Bi_2_Te_3_ thickness. The overall exponential decrease of *J* with increasing *n* is consistent with the observed reduction in coercivity, indicating that both parameters coherently reflect the weakening of interlayer coupling. Importantly, the magnitude of our experimentally derived *J* values is comparable to those reported previously for MnBi_2_Te_4_(Bi_2_Te_3_)*_n_* superlattices [48], further supporting the reliability of our estimation.

**FIGURE 5:**
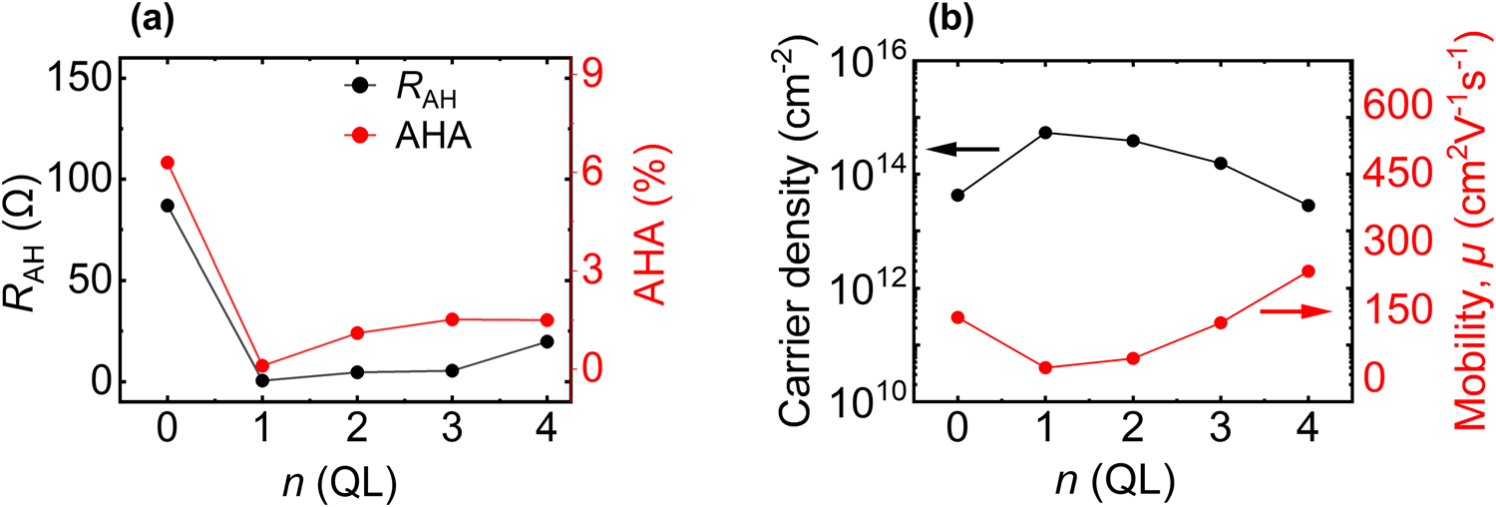
Dependence of transport behaviour on Bi_2_Te_3_ spacer thickness in 1 SL MnBi_2_Te_4_/*n* QL Bi_2_Te_3_/1 SL MnBi_2_Te_4_ heterostructure. (a) Variation of anomalous Hall resistance (*R*_AH_) and anomalous Hall angle (AHA) with the TI thickness in the heterostructures measured at 3 K, highlighting transport tunability with interlayer separation. (b) The carrier density (left axis) and mobility (right axis) measured at 3 K as a function of Bi_2_Te_3_ quintuple layer (QL) thickness.

Figure 5a illustrates the dependence of *R*_AH_ and the anomalous Hall angle (AHA) on Bi_2_Te_3_ thickness (*n* = 0 to 4), measured at 3 K. Both *R*_AH_ and AHA exhibit a non‐monotonic trend on spacer thickness, revealing insights into the magnetic and transport coupling across the TI layer. The maximum *R*_AH_ was observed in the pristine 2 SL MnBi_2_Te_4_ film (*n* = 0), reaching 87 Ω. Upon insertion of a single QL of Bi_2_Te_3_, the *R*_AH_ dropped sharply to only 0.5 Ω, indicating a strong suppression of the anomalous Hall effect due to the disrupted exchange coupling. With further increase in QLs, *R*_AH_ partially recovered to 4.64 Ω (*n* = 2), 5.38 Ω (*n* = 3), and 20 Ω (*n* = 4).

A very similar trend, like *R*_AH_, is observed in AHA. The AHA is quantified as the efficiency of transverse charge deflection due to the anomalous Hall effect, and is defined as: AHA (%) = $\frac{\Delta \sigma_{xy}^{AH}}{\Delta \sigma_{xx}}$ ×100, where $\Delta \sigma_{xy}^{AH}$ and Δσ_*xx*_ are anomalous Hall conductance and longitudinal conductance, respectively [52, 53, 54]. The highest AHA value of 6.3% occurs at *n* = 0, decreasing to 0.5% at *n* = 1, then rising to 1.1%, 1.52% and 1.5% for *n* = 2, 3, and 4, respectively.

Interestingly, while Figure 4 shows that the interlayer coupling weakens with increasing *n* as intuitively expected, both *R*_AH_ and AHA increase from *n* = 1 to 4. This behaviour arises because increasing the Bi_2_Te_3_ thickness facilitates the establishment of FM order in the MnBi_2_Te_4_ layers, leading to a more well‐ordered FM state with reduced spin fluctuations, which in turn enhances the anomalous Hall response [55]. This apparent increase in the Hall signal can also be explained due to the decreased carrier densities. As shown in Figure 5b, the carrier density slightly decreases with increasing *n* between 1 and 4, which can be attributed to the progressively reduced coupling between the top and bottom MnBi_2_Te_4_ layers. Previous studies have shown that thinner Bi_2_Te_3_ or MnBi_2_Te_4_‐Bi_2_Te_3_ multilayers exhibit stronger inter‐surface coupling [56, 57], which enhances bulk‐like carrier contributions. As the film thickness increases, this coupling weakens, shifting the Fermi level closer to the bulk gap and thereby lowering the carrier density [56]. In addition, thicker Bi_2_Te_3_ or MnBi_2_Te_4_‐Bi_2_Te_3_ multilayers typically exhibit improved structural quality and stoichiometry, further suppressing bulk carrier channels. Therefore, since *R*_xy_ is inversely proportional to carrier density, this reduction amplifies the anomalous Hall response. Additionally, the AHA, being a conductivity ratio, also benefits from lower carrier density [58]. These considerations are fully consistent with our results and provide a clear rationale for the observed reduction in carrier density. In contrast, the mobility showed an inverse trend: for *n* = 0, the mobility was 135.67 cm^2^V^−1^s^−1^, which dropped to 24.87 cm^2^V^−1^s^−1^ for *n* = 1, then increased with *n* and reached a maximum of 237.28 cm^2^V^−1^s^−1^ for *n* = 4. This inverse relationship between carrier density and mobility suggests that enhanced scattering in higher‐density samples reduces carrier mobility [59]. These transport parameters are comparable with prior reports on magnetically doped TI systems [10, 42, 60], confirming the reliability of our growth and fabrication methods. The detailed comparisons of temperature dependent *R*_AH_ and AHA among all heterostructures are provided in Figure S10.

## 3 Conclusion

In summary, by combining MnBi_2_Te_4_ with Bi_2_Te_3_ in a tuneable van der Waals sandwich architecture, we reveal a controllable transition between AFM and FM interlayer ordering, accompanied by distinct changes in transport behaviour. These findings establish a platform for designing spintronic and quantum devices where magnetism and topology can be finely modified via structural engineering rather than chemical doping. However, although these heterostructures possess large magnetic gaps and are theoretically predicted to host the QAHE [25, 33], they exhibit a high electron carrier density (∼10^14^ cm^−^^2^). This places the Fermi level in the bulk conduction band rather than inside the magnetic gap [25]. Achieving QAHE in these heterostructures therefore requires tuning the Fermi level into the magnetic gap, which in turn necessitates reducing the n‐type doping. A natural direction for future research is to suppress this unintentional n‐type doping, for example by employing (Bi_1‐x_Sb_x_)_2_Te_3_ (BST) spacers, where the Bi–Sb ratio enables continuous tuning of the carrier density from n‐type to p‐type [61]. Such carrier‐compensation and band‐alignment control have been crucial for realizing QAHE in dilute magnetic TIs [10, 41], and applying similar Fermi‐level‐engineering strategies may provide a promising pathway for achieving QAHE in MnBi_2_Te_4_‐Bi_2_Te_3_ based heterostructures. Therefore, future efforts may build upon this framework to achieve quantum anomalous Hall phases at elevated temperatures and to probe magnetic proximity effects in precisely engineered quantum materials.

## 4 Experimental Methods

### 4.1 MnBi_2_Te_4_/Bi_2_Te_3_/MnBi_2_Te_4_ Heterostructure Growth

See Supporting Information for detailed growth procedure.

### 4.2 Transport Measurements

Transport measurements, using the van der Pauw geometry, were performed in a Quantum Design Physical Property Measurement System (PPMS). Raw data corresponding to *R*_xx_ and *R*_xy_ were symmetrized and anti‐symmetrized, respectively, to eliminate longitudinal contributions in *R*_xy_ and transverse contributions in *R*_xx_ arising from contact misalignment.

### 4.3 SQUID Measurements

Magnetometry measurements were performed in a Quantum Design Magnetic Property Measurement System (MPMS) equipped with SQUID (superconducting quantum interference device). Raw data represented the total magnetic moment as a function of externally applied perpendicular magnetic field. Raw data was background subtracted to isolate the intrinsic magnetic response of the film from the diamagnetic response of the STO substrate.

### 4.4 XRD Measurements

XRD measurements were performed using a Bruker D8 Advance employing CuK_𝛂_ radiation (λ = 1.5406 Å).

### 4.5 TEM and EELS Measurements

Cross sectional TEM sample was prepared using in situ lift‐out method. To avoid charging problems, the sample was coated with a thin carbon film. Prior to sample preparation, a 300 nm thick Pt protection layer was deposited using the electron‐beam deposition facility of the Thermo Fisher Aquilos2 dual‐beam FIB system. After transferring onto a copper grid TEM lamella was prepared using 30 keV Ga FIB milling with 3 nA and 300 pA beam currents. Finally, it was cleaned using low energy FIB milling (5 keV and 77 pA beam current). TEM sample were examined using FEI Tecnai TF30 transmission electron microscope operated at 200 kV. EELS measurements were conducted in STEM mode, with 1 nm probe beam diameter, convergence semi‐angle of 2.2 mrad, and a collection semi‐angle of 16 mrad using Gatan GIF QuantumTM 965 energy filter.

### 4.6 Statistical Analysis

Transport and magnetization measurements were performed under stable temperature and magnetic field conditions to ensure reliability. For each heterostructure (*n* = 0–4), a single representative sample was measured, and reproducibility was confirmed through repeated field and temperature sweeps. Noise was minimized using standard calibration modes and averaging multiple points during field sweeps. No data transformation or normalization was applied. Statistical analysis and plotting were carried out with OriginPro 2024.

## Author Contributions

The concept of the MnBi_2_Te_4_/Bi_2_Te_3_/MnBi_2_Te_4_ heterostructure was originally proposed by M.T.E., forming the basis of this study. E.H. received MBE training from G.L.C., and then designed and executed the experiments under the guidance of M.T.E., M.S.F., and G.L.C. E.H. prepared the samples with support from G.L.C., Q.L., M.T.H.B., J.B., M.Z., and M.G. Sample characterization via X‐ray diffraction was performed by E.H., with assistance from G.L.C. Transport and magnetometry measurements were carried out by E.H., with experimental support from G.L.C., M.G., and K.X. TEM and EELS measurements were carried out by S.R. E.H. wrote the manuscript, and M.T.E., M.S.F. and G.L.C. contributed to its review and editing. The authors thank W. Zhao for valuable discussions and Y. Zhao for sharing the STO treatment recipe.

## Conflicts of Interest

The authors declare no conflicts of interest.

## Supporting information




**Supporting File**: advs73618‐sup‐0001‐SuppMat.pdf.

## Data Availability

The data that support the findings of this study are available from the corresponding author upon reasonable request.
