# Supplementary material for: Ferromagnetic Interlayer Exchange Coupling in Magnetic Topological Insulator Sandwich Heterostructures
Source: Adv Sci (Weinh). 2026 Jan 27;13(14):e14562. doi: 10.1002/advs.202514562 (PMC12970225; doi:10.1002/advs.202514562)
Supplement: Supplementary file 1 — Supporting File: advs73618‐sup‐0001‐SuppMat.pdf. [file ADVS-13-e14562-s001.pdf]

Supporting Information for

Ferromagnetic Interlayer Exchange Coupling in Magnetic Topological Insulator Sandwich Heterostructures

Enayet Hossain[1,2,3,*], Grace L. Causer[1,2], Qile Li[1,2], Sergey Rubanov[4], Kaijian Xing[1], James Blyth[1,2], Mohammad T. H. Bhuiyan[1], Mengting Zhao[1,2], Matthew Gebert[1,2], Michael S. Fuhrer[1,2], and Mark T. Edmonds[1,2,*]

[1]School of Physics and Astronomy, Monash University, Clayton, Victoria, Australia

[2]ARC Centre of Excellence for Future Low-Energy Electronics Technologies (FLEET), Monash University, Clayton, Victoria, Australia

[3]Department of Physics, University of Dhaka, Dhaka-1000, Bangladesh

[4]Ian Holmes Imaging Centre (IHIC), Bio21 Institute, University of Melbourne, Victoria, Australia

Corresponding authors: enayet.hossain@du.ac.bd
enayet.hossain@monash.edu
mark.edmonds@monash.edu

Contents:

Section I. **$MnBi_2Te_4/Bi_2Te_3/MnBi_2Te_4$ heterostructure growth and structural characterization**

Section II. **Temperature dependent sheet resistance (R_{xx})**

Section III. **Transport measurements of 2SL $MnBi_2Te_4$ ($n = 0$)**

Section IV. **Two-component anomalous Hall (AH) in 2SL $MnBi_2Te_4$**

Section V. **Magnetic-field-dependent Hall resistance across all heterostructures at 3 K**

Section VI. **Magnetic-field-dependent Hall and anomalous Hall for n = 1–3**

Section VII. **Extracted transport parameters for all heterostructures**

Section I. $MnBi_2Te_4/Bi_2Te_3/MnBi_2Te_4$ heterostructure growth and structural characterization

1 SL $MnBi_2Te_4$/*n* QL Bi_2Te_3/1 SL $MnBi_2Te_4$ heterostructures (n = 0 to 4) were grown on strontium titanate ($SrTiO_3$), STO (111) substrates. Before growth, the STO is boiled in deionized water (around 90°C) for 1 hour. After that, in order to achieve a passivated and atomically flat surface, the STO is annealed in O_2 flowing atmosphere at 980°C.[1,2]

After the O_2 annealing, STO samples are immediately loaded into ultra-high vacuum in the MBE chamber. The STO is then annealed at 600°C for two hours to further improve surface quality. This process yields a clear reflection high-energy electron diffraction (RHEED) pattern, indicating a well-prepared and atomically flat substrate, consistent with literature[2,3] (**Figure S1**(a)). For the heterostructure growth, 99.999% Te, 99.95% Bi and 99.9% Mn were evaporated using MBE component effusion cells, with material deposition rates calibrated using a quartz crystal microbalance (QCM).

The bottom and top 1 SL $MnBi_2Te_4$ layers in the 1 SL $MnBi_2Te_4$/*n* QL Bi_2Te_3/1 SL $MnBi_2Te_4$ heterostructure were grown by first depositing 1QL of Bi_2Te_3 and then a bilayer of MnTe, which is known to spontaneously rearrange into 1 SL $MnBi_2Te_4$.[4] The middle Bi_2Te_3 part of the heterostructure was grown for the desired number of QLs. The time to grow each Bi_2Te_3 QL was determined to be 170 s, based on the distinct RHEED oscillations indicating layer-by-layer growth shown in Figure S1(d). The sample was annealed in Te flux for 5 mins after the bottom 1 SL $MnBi_2Te_4$ was grown and then again after the Bi_2Te_3, and once more when the heterostructure was complete, in order to minimize Te deficiency and improve crystallinity.[2,4] During the whole growth, the substrate temperature was maintained at around 250°C. After growth, films were capped with a 10 nm amorphous Te layer to prevent oxidation during air exposure and subsequent transfer to the low-temperature cryostats for electrical transport and magnetometry measurements.

The RHEED patterns of 2 SL $MnBi_2Te_4$ film ((Figure S1(b)) and 1 SL $MnBi_2Te_4$/4 QL Bi_2Te_3/1 SL $MnBi_2Te_4$ heterostructure in Figure S1(c) show a single set of sharp streaks due to the almost identical in-plane lattice constants of Bi_2Te_3 (a = 4.38 Å)[5] and $MnBi_2Te_4$ (a = 4.3304 Å),[6] indicating smooth surface morphology and high crystallinity.[1] The structure was confirmed by X-ray diffraction (XRD) as shown in Figure S1(e), where distinct (00l) peaks of Bi_2Te_3 and $MnBi_2Te_4$ confirm proper phase formation of the layered crystal structure along the c-axis.[7–

[10] The absence of $MnBi_2Te_4$ peaks at lower 2θ is attributed to its ultrathin thickness compared to the 4 QL Bi_2Te_3 spacer.

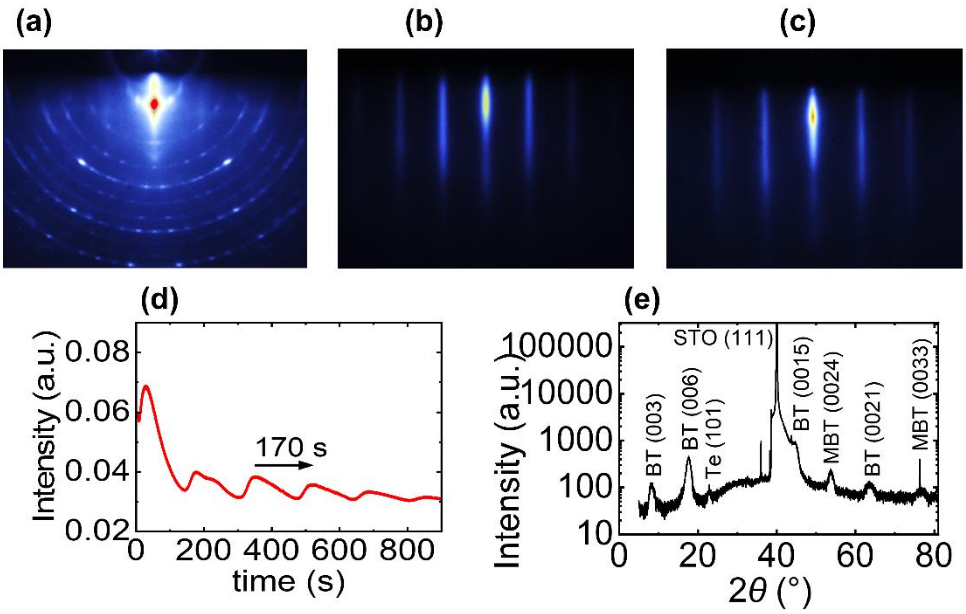

Figure S1. MBE growth and structural characterization of 1 SL $MnBi_2Te_4$/*n* QL Bi_2Te_3/1 SL $MnBi_2Te_4$ heterostructure. RHEED patterns of (a) the pristine STO (111) substrate, (b) 2 SL $MnBi_2Te_4$ and (c) 1 SL $MnBi_2Te_4$/4 QL Bi_2Te_3/1 SL $MnBi_2Te_4$ heterostructure. (d) RHEED intensity oscillations, exhibiting layer-by-layer growth of Bi_2Te_3 on STO (111) substrate. (e) X-ray diffraction (XRD) pattern of 1 SL $MnBi_2Te_4$/4 QL Bi_2Te_3/1 SL $MnBi_2Te_4$

To directly validate the interlayer interface quality, we have carried out cross-sectional high-resolution TEM together with spatially resolved electron energy loss spectroscopy (EELS). As shown in Figure S2(a), a thin Te buffer layer is clearly visible at the STO (111) interface, originating from the initial Te pre-deposition step routinely employed in MBE to promote smooth van der Waals nucleation. The interface between STO and the Te buffer layer is atomically sharp, with no detectable intermixing or interfacial reaction, indicating a clean and well-prepared substrate surface.

Beyond this expected Te buffer layer, the transition into the bottom 1 SL $MnBi_2Te_4$ layer is also atomically abrupt, and no amorphous or chemically disordered region is observed. Then across the heterostructure, the lattice fringes of the bottom $MnBi_2Te_4$ layer, the 4 QL Bi_2Te_3 spacer, and the top 1 SL $MnBi_2Te_4$ layer remain continuous with minimal surface roughness, demonstrating the atomic-level flatness of each interface.[11,12] To further verify that Mn is only

present in the 1 SL $MnBi_2Te_4$ layers, we performed EELS line-profile analysis (Figure S2(b)). The Mn-L_3 edge is distinctly observed in both the top and bottom 1 SL $MnBi_2Te_4$ layers but is completely absent in the 4 QL Bi_2Te_3 spacer,[13] confirming the correct chemical stacking sequence in the heterostructure. The complete suppression of the Mn signal in the Bi_2Te_3 region verifies that no Mn diffusion or interlayer intermixing occurred during growth. These combined TEM and EELS results directly demonstrate the high-quality of our heterostructure interfaces.

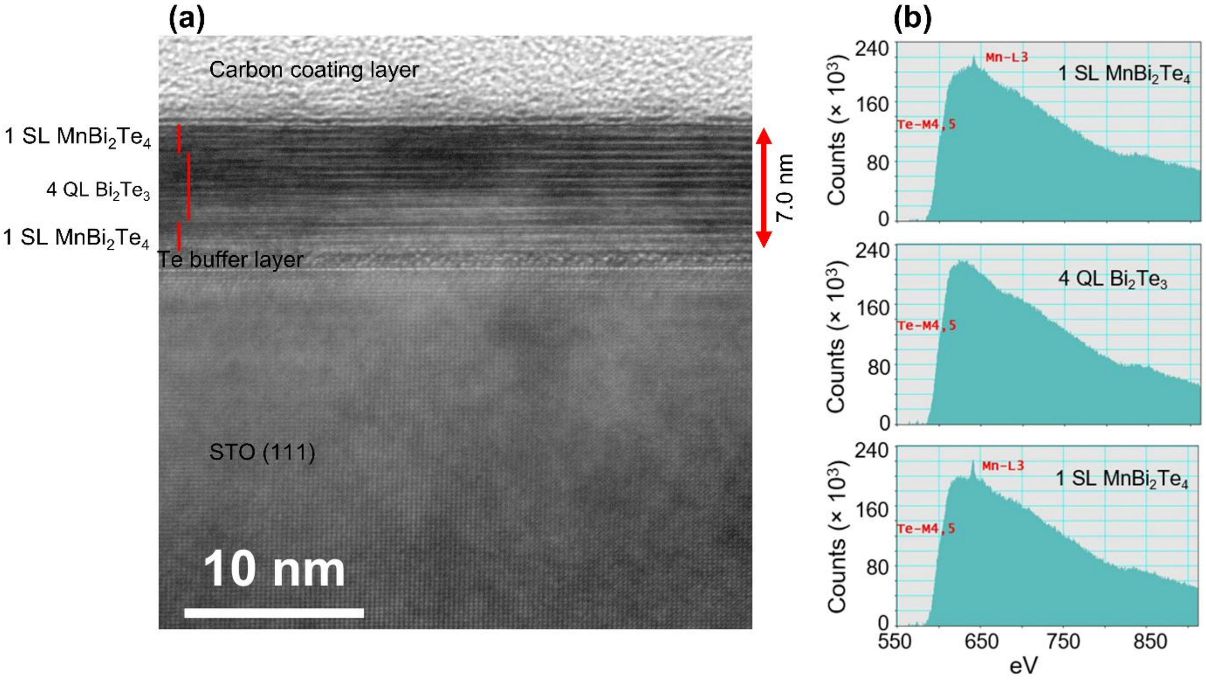

Figure S2. Cross-sectional high-resolution TEM and EELS of 1 SL $MnBi_2Te_4$/4 QL Bi_2Te_3/1 SL $MnBi_2Te_4$ heterostructure. (a) The TEM image shows that the lattice fringes remain continuous across each interface, with no visible intermixing or amorphous layer, indicating that the interfaces are atomically abrupt and well-defined. (b) EELS profiles for the top 1 SL $MnBi_2Te_4$ (upper panel), the 4 QL Bi_2Te_3 spacer (middle panel), and the bottom 1 SL $MnBi_2Te_4$ (lower panel). The Mn–L_3 edge is clearly visible in the two $MnBi_2Te_4$ layers but absent in the 4 QL Bi_2Te_3 region, confirming that Mn does not diffuse into the Bi_2Te_3 spacer.

Section II. Temperature dependent sheet resistance (R_{xx})

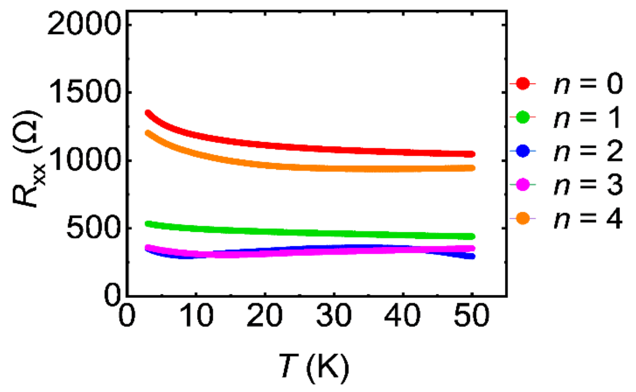

Figure S3. Temperature dependence of the longitudinal sheet resistance (R_{xx}) for heterostructures with varying Bi_2Te_3 thickness (n = 0 to 4).

Figure S3 presents the temperature dependence of the longitudinal sheet resistance R_{xx} (T) for a series of 1 SL $MnBi_2Te_4$/n QL Bi_2Te_3/1 SL $MnBi_2Te_4$ heterostructures with n = 0 to 4 at zero magnetic field. Overall, in all cases, R_{xx} decreases with increasing temperature up to ~15 K, followed by a weak saturation at higher temperatures, indicating thermally activated transport at low temperatures, while a temperature-independent conduction mechanism becomes dominant at elevated temperatures. The absence of any kink-like feature in the R_{xx} across all heterostructures (n = 1–4) indicate an interlayer ferromagnetic phase transition rather than antiferromagnetic. While such features are typically expected near the Neel temperature in antiferromagnets, their absence in 2 SL $MnBi_2Te_4$ can be attributed to weak interlayer exchange coupling in ultrathin films.[2] This weak coupling may result in a less pronounced change in resistance at the Neel temperature, making the kink or peak feature less visible in the R_{xx} data.

Section III. Transport measurements of 2SL $MnBi_2Te_4$ (n = 0)

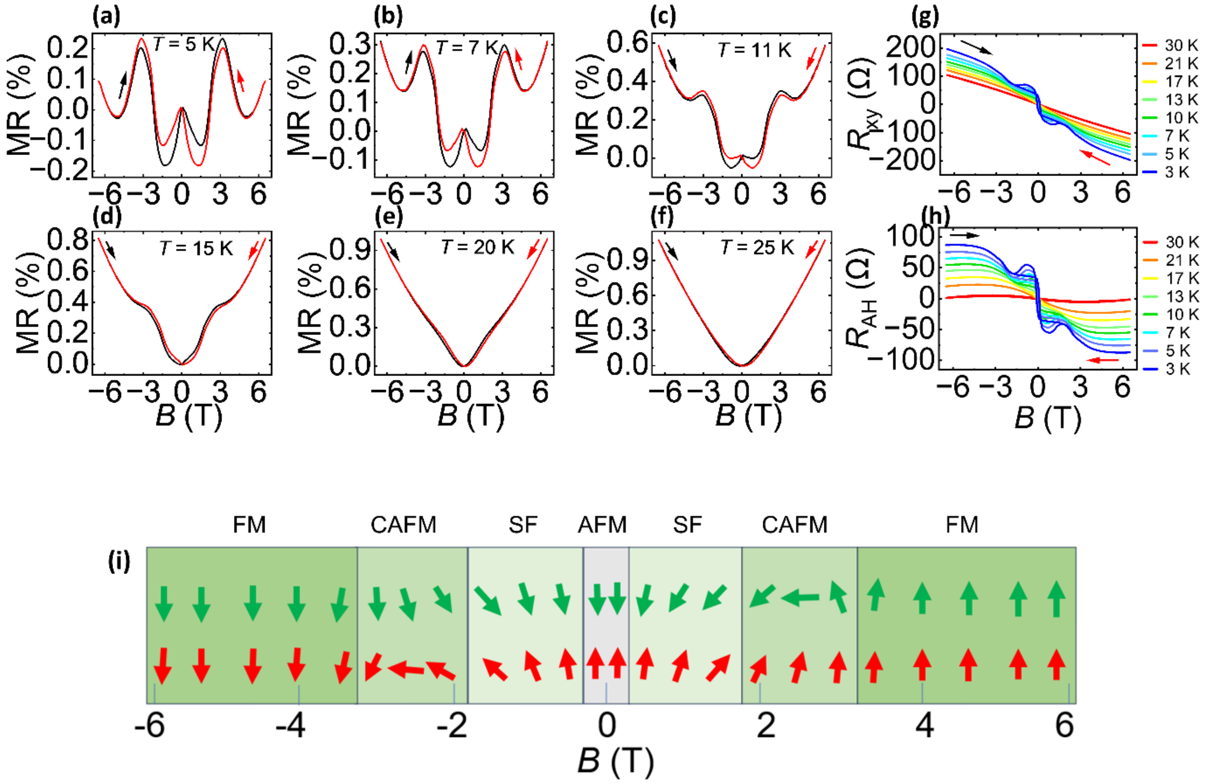

Figure S4. Magnetotransport response in 2 SL $MnBi_2Te_4$. (a–f) Magnetoresistance, MR measured between 5 and 25 K. (g) Hall resistance, R_{xy}, and (h) Anomalous Hall resistance, R_{AH} of the 2 SL $MnBi_2Te_4$ film. Both MR and R_{xy} data clearly indicates the features of spin-flop transitions governed by interlayer antiferromagnetic coupling. Black and red arrows indicate increasing and decreasing field directions, respectively. (i) Schematic showing how complex spin structure in 2 SL $MnBi_2Te_4$ evolves in external magnetic field. The schematic presented here is adapted from the magnetic structure representation for 3 SL $MnBi_2Te_4$ in Zhu et al.,[9] but has been independently created to depict the spin structure of 2 SL $MnBi_2Te_4$, with modifications to reflect the specific characteristics of 2 SL system.

At low temperatures ($T \leq 15$ K) in Figure S4, the MR exhibits a characteristic multi-step structure, which arises from the sequential spin-flop transition and CAFM state under applied magnetic field as discussed in the main text. In contrast, at $T \geq 20$ K, the MR evolves into a smooth, monotonic, V-shaped or parabolic profile without any signatures of spin-flop or

canting, closely resembling the response observed in our FM-coupled 1 SL $MnBi_2Te_4$/*n* QL Bi_2Te_3/1 SL $MnBi_2Te_4$ heterostructures. The gradual disappearance of the CAFM-related features indicates that thermal fluctuations progressively weaken the interlayer AFM coupling, driving the system toward a more FM-like interlayer response.

Figures S4(g) and S4(h) show the Hall and anomalous Hall responses of the 2 SL $MnBi_2Te_4$ sample. Consistent with the MR evolution, the Hall traces exhibit a clear temperature-dependent change in magnetic behaviour. At low temperatures, the nonlinear R_{xy}–B curves and the presence of a complex hysteretic anomalous Hall component reflect the CAFM state. As the temperature increases, the anomalous Hall contribution gradually weakens, and by $T \geq 21$ K, the R_{xy} response becomes hysteresis-free and nearly linear across the entire field range, indicating that the system has entered a paramagnetic regime where the interlayer magnetic correlations are effectively suppressed.

Section IV. Two-component anomalous Hall (AH) in 2SL $MnBi_2Te_4$ (n = 0)

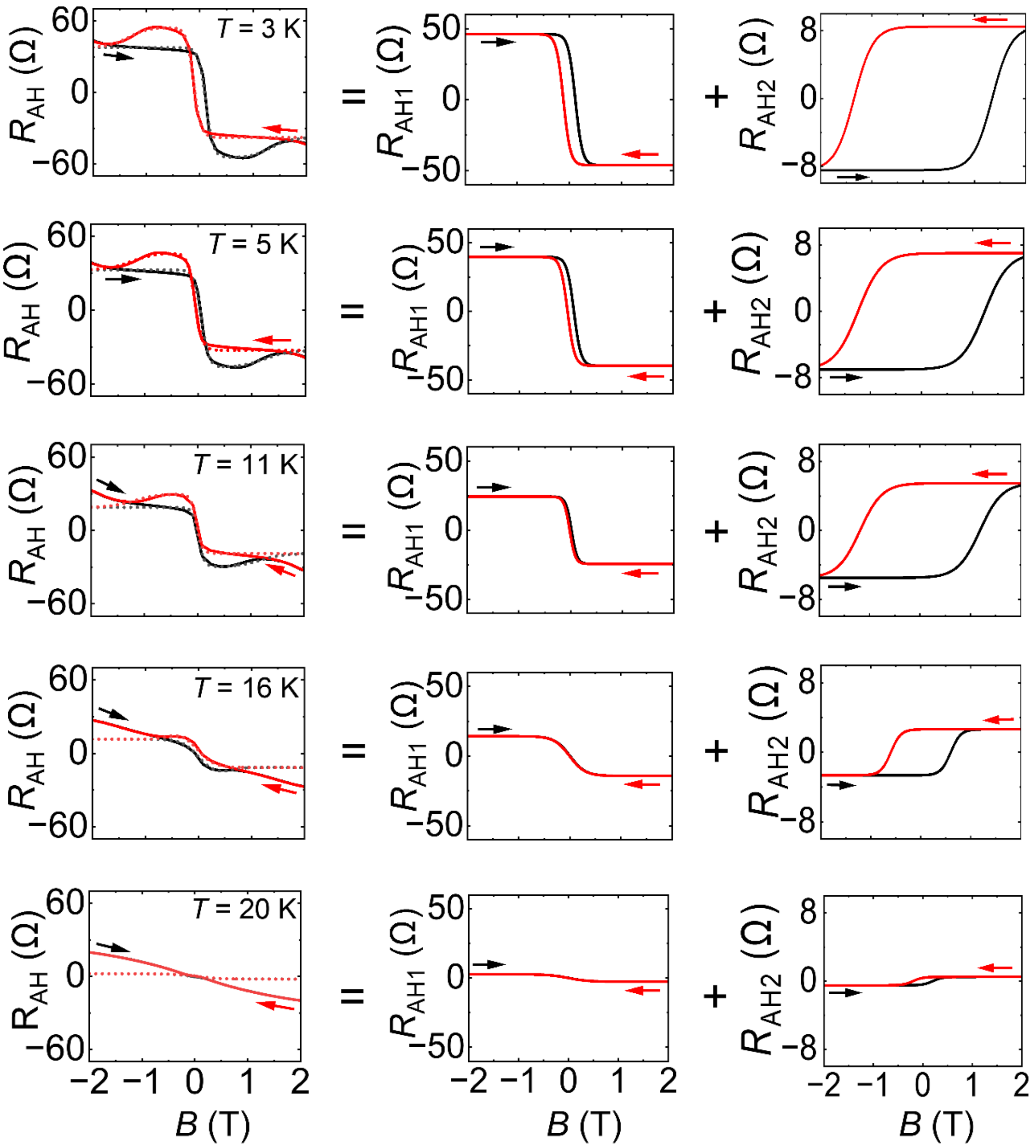

Figure S5. The decomposition of the anomalous Hall resistance (R_{AH}) into two components R_{AH1} and R_{AH2} for 2 SL $MnBi_2Te_4$ measured between 3 and 20 K. Black and red arrows indicate increasing and decreasing field directions, respectively. The dashed line in R_{AH} represents the fitted results.

At 3 K in Figure S5, the extracted anomalous Hall resistance (R_{AH}) shows a non-square hysteresis loop, indicative of two anomalous Hall components, R_{AH1} and R_{AH2}, contributing to

the overall R_{AH} behaviour and is expressed as $R_{AH} = R_{AH1} + R_{AH2}$. This behaviour is modelled using a two-component fit of the form:

$R_{AH}(B) = R_1 \tanh(w_1 (B - B_{C1})) + R_2 \tanh(w_2 (B - B_{C2}))$.[2,9]

where, B_{C1} and B_{C2} are the coercive fields corresponding to AH components R_{AH1} and R_{AH2}; R_1, R_2 are their amplitudes; and w_1, w_2 are fitting parameters controlling switching sharpness.

Over the full temperature range (3 K to 30 K) as shown in Figure S6, the R_{AH2} at zero field, denoted by $R_{AH2(0)}$, remains positive and is attributed to the AFM and canted AFM interlayer coupling in the 2 SL $MnBi_2Te_4$ film. In contrast, $R_{AH1(0)}$ is consistently negative across all temperatures, and may originate from intrinsic ferromagnetic contributions within the $MnBi_2Te_4$ film itself, potentially due to localized FM domains or defects which induce a net magnetization.[2,14] So, the opposite polarity of R_{AH1} and R_{AH2} indicates the opposite magnetic coupling between the two regions that create anomalous Hall signal.

The variation of $R_{AH1(0)}$ and $R_{AH2(0)}$ as a function of temperature is presented in Figure S6. Notably, $R_{AH1(0)}$ vanishes at approximately 21 K, in good agreement with literature.[2]

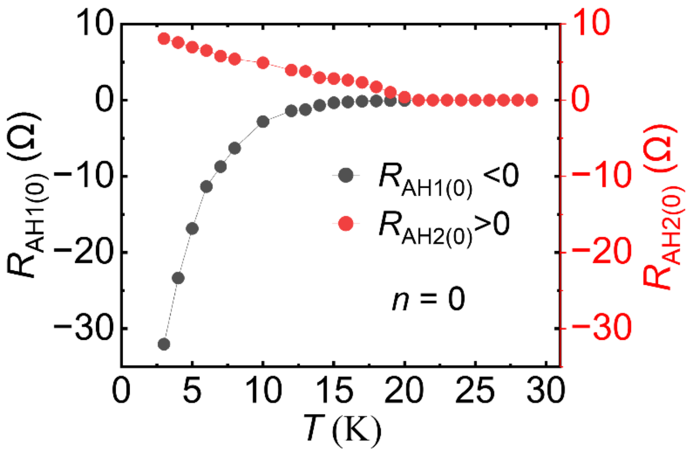

Figure S6. Temperature dependence of R_{AH1} and R_{AH2} of 2 SL $MnBi_2Te_4$ film.

Section V. Magnetic-field-dependent Hall resistance across all heterostructures at 3 K

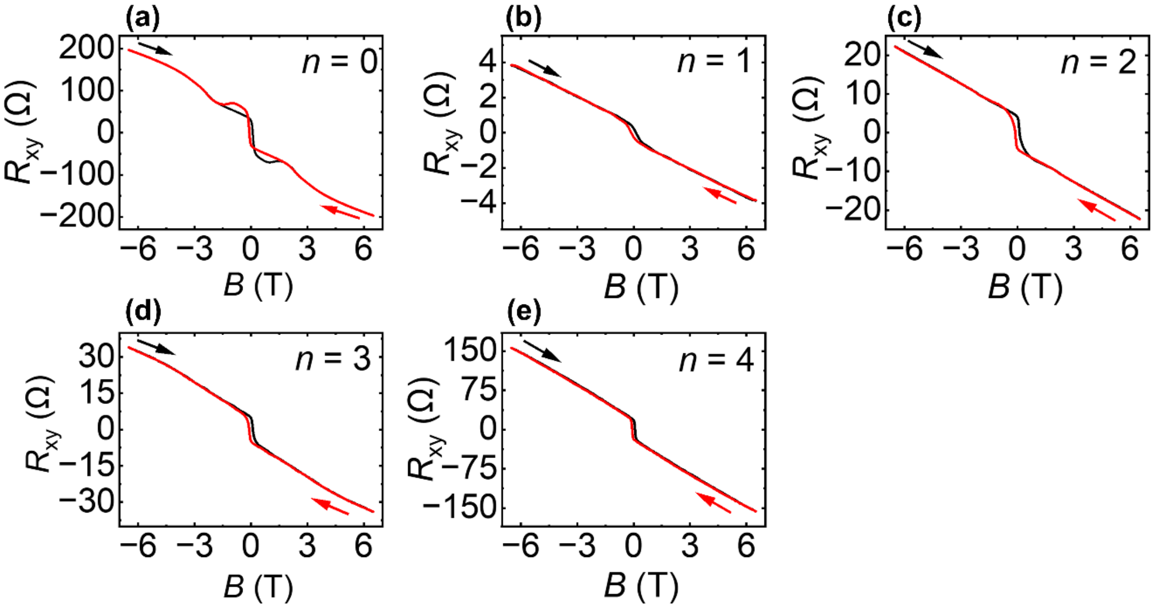

Figure S7. Magnetic field dependence of the Hall resistance measured at 3 K for (a) 2 SL $MnBi_2Te_4$ (n = 0), and (b-e) heterostructures with n = 1–4 in the 1 SL $MnBi_2Te_4$/n QL Bi_2Te_3/1 SL $MnBi_2Te_4$ series. The 2 SL $MnBi_2Te_4$ film exhibits a spin-flop transition, indicative of interlayer AFM coupling. In contrast, the heterostructures with Bi_2Te_3 spacers display a pronounced hysteresis loop around zero field, followed by a completely linear response across the entire field range, confirming a transition to interlayer FM ordering. Black and red arrows indicate increasing and decreasing field directions, respectively.

Although the polarity of R_{AH1} and R_{AH2} in 2 SL $MnBi_2Te_4$ are opposite with different coercivity, in the 1 SL $MnBi_2Te_4$/n QL Bi_2Te_3/1 SL $MnBi_2Te_4$ heterostructure (n = 1–4), the interlayer coupling is FM with a single coercive field for each heterostructure. This contrasting magnetic switching behaviours arise from fundamentally different interlayer coupling mechanisms.

When two $MnBi_2Te_4$ layers are directly stacked without a Bi_2Te_3 spacer (n = 0 case), they exhibit AFM interlayer coupling, consistent with the intrinsic A-type AFM order of bulk $MnBi_2Te_4$. This AFM alignment arises from Te-mediated superexchange between adjacent septuple layers, which energetically favours antiparallel Mn–Mn alignment following the Goodenough–Kanamori–Anderson rules.[15–18] This AFM coupling results in the two $MnBi_2Te_4$

layers having opposite anomalous Hall resistance (sign reversal) and distinct coercivities, consistent with their antiparallel magnetic configuration.

However, once a Bi_2Te_3 spacer is inserted, the interlayer coupling changes from AFM to FM. This is clearly evidenced in our heterostructure by the emergence of a square-shaped anomalous Hall hysteresis and the absence of spin-flop transitions.

The Bi_2Te_3 spacer interrupts the superexchange pathway, effectively suppressing the intrinsic AFM interlayer coupling. In this regime, long-range dipolar interactions and proximity-induced exchange coupling through the spin-polarized surface states of Bi_2Te_3 become dominant, both favouring a parallel alignment of magnetic moments in $MnBi_2Te_4$ layers. As a result, the two layers switch simultaneously, exhibiting similar coercivities and the same sign of anomalous Hall resistance.[19–21]

Section VI. Magnetic-field-dependent Hall and anomalous Hall for $n = 1 - 3$

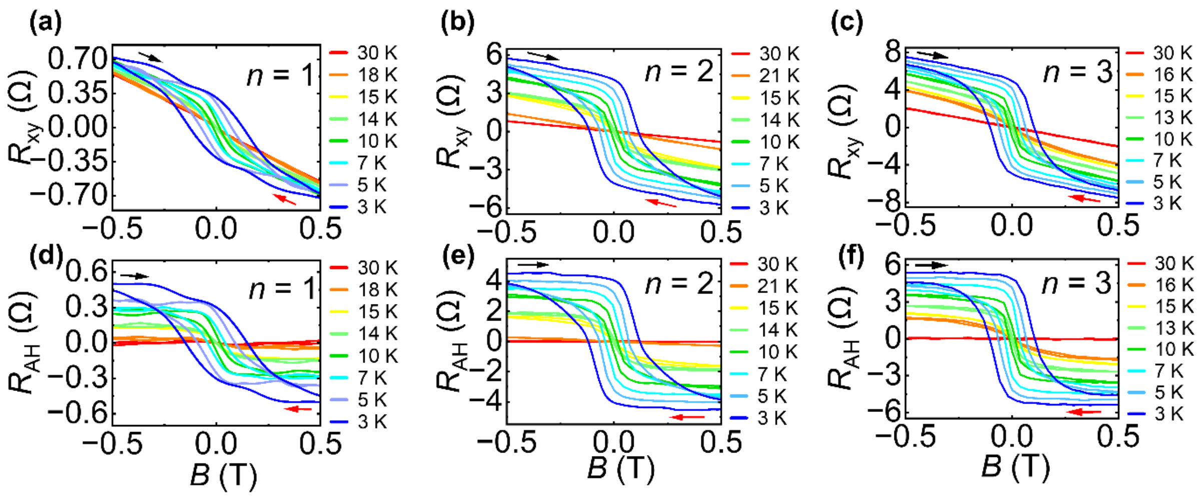

Figure S8. (a–c) Hall resistance, R_{xy} and (d–f) Anomalous Hall resistance, R_{AH} for 1 SL $MnBi_2Te_4$/*n* QL Bi_2Te_3/1 SL $MnBi_2Te_4$ heterostructures, with (a, d) $n = 1$, (b, e) $n = 2$ and (c, f) $n = 3$, measured between 3 K and 30 K. The R_{AH} curves were obtained by subtracting the linear ordinary Hall background from R_{xy}. Black and red arrows indicate increasing and decreasing field directions, respectively.

Section VII. Extracted transport parameters for all heterostructures

Figure 4(c) in the main manuscript shows the interlayer magnetic coupling strength (J) as a function of Bi_2Te_3 QL thickness. The coupling strength was calculated using the formula:[22]

$$J = \frac{1}{S} g\mu_B \frac{B_c^S}{z} \quad (1)$$

Here, $S = \frac{5}{2}$ is the spin quantum number of Mn^{2+}, μ_B = Bohr magneton (9.274×10^{-24} J/T), B_c^s is the critical saturation field, defined as the magnetic field at which the magnetization begins to approach its saturated value after the hysteresis loop, z = 6 denotes the number of nearest Mn neighbours in the adjacent layers and, g = 2 is the Lande g-factor.

In our heterostructures, magnetometry measurements were only available for the 1 SL $MnBi_2Te_4$/4 QL Bi_2Te_3/1 SL $MnBi_2Te_4$ sample. For this sample, we determined B_c^s independently from both the magnetization loop and the field dependence of the anomalous Hall resistance. The two extracted values of J were found to be in good agreement, validating the use of the transport-derived critical saturation field as a reliable measure. We therefore used the transport data to extract B_c^s and hence J for the remaining heterostructures (n = 1–4). A representative anomalous Hall curve for n = 4, illustrating the determination of B_c^s is provided in Figure S9.

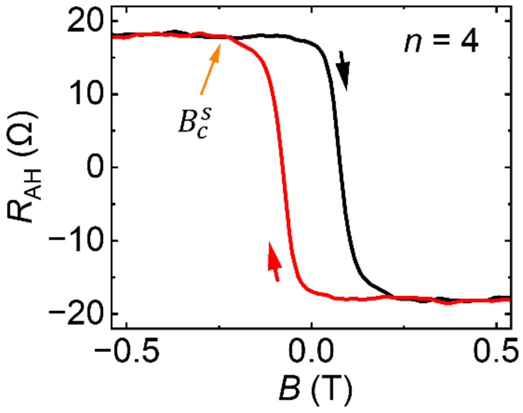

Figure S9. Field dependence of the anomalous Hall resistance R_{AH} for the1 SL $MnBi_2Te_4$/4 QL Bi_2Te_3/1 SL $MnBi_2Te_4$ heterostructure measured at 3 K. The critical saturation field B_c^s is determined from the field at which R_{AH} (B) enters the saturation regime.

The anomalous Hall resistance, R_{AH} is higher at 3 K in each heterostructure and decreases monotonically with temperature as shown in Figure S10(a). For the 2 SL $MnBi_2Te_4$ (n = 0) film, R_{AH} vanishes near 25 K. Among the heterostructures, n = 1 exhibits the lowest R_{AH}, while n = 2 and 3 show slightly enhanced values, and the n = 4 structure yields the highest R_{AH}, despite its weaker interlayer magnetic ordering. This behaviour is attributed to the significantly lower carrier density in the n = 4 sample, which amplifies the anomalous Hall response.[23]

In Figure S10(b), anomalous Hall angle (AHA) decreases steadily with temperature for all heterostructures, consistent with the suppression of interlayer magnetic ordering. The trends observed in both R_{AH} and AHA are also consistent with the carrier density-dependent behaviour discussed in the main manuscript.

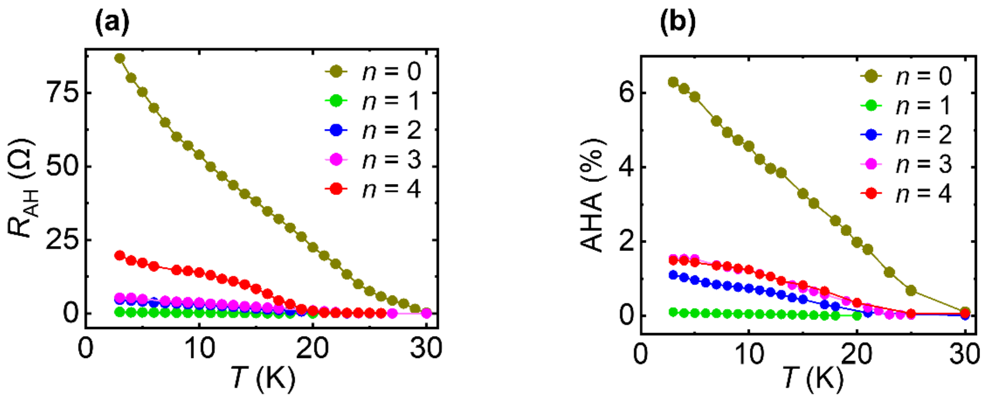

Figure S10. Transport and anomalous Hall parameters of 1 SL $MnBi_2Te_4$/n QL Bi_2Te_3/1 SL $MnBi_2Te_4$ heterostructures with n = 0–4. (a) The temperature dependence of anomalous Hall resistance (R_{AH}) for all samples. (b) Anomalous Hall angle (AHA) as a function of temperature.

References

(1) Zhao, Y.-F.; Zhang, R.; Mei, R.; Zhou, L.-J.; Yi, H.; Zhang, Y.-Q.; Yu, J.; Xiao, R.; Wang, K.; Samarth, N.; Chan, M. H. W.; Liu, C.-X.; Chang, C.-Z. Tuning the Chern Number in Quantum Anomalous Hall Insulators. *Nature* **2020**, *588* (7838), 419–423. https://doi.org/10.1038/s41586-020-3020-3.

(2) Zhao, Y.-F.; Zhou, L.-J.; Wang, F.; Wang, G.; Song, T.; Ovchinnikov, D.; Yi, H.; Mei, R.; Wang, K.; Chan, M. H. W.; Liu, C.-X.; Xu, X.; Chang, C.-Z. Even–Odd Layer-Dependent Anomalous Hall Effect in Topological Magnet $MnBi_2 Te_4$ Thin Films. *Nano Lett.* **2021**, *21* (18), 7691–7698. https://doi.org/10.1021/acs.nanolett.1c02493.

(3) Jiang, J.; Xiao, D.; Wang, F.; Shin, J.-H.; Andreoli, D.; Zhang, J.; Xiao, R.; Zhao, Y.-F.; Kayyalha, M.; Zhang, L.; Wang, K.; Zang, J.; Liu, C.; Samarth, N.; Chan, M. H. W.; Chang, C.-Z. Concurrence of Quantum Anomalous Hall and Topological Hall Effects in Magnetic Topological Insulator Sandwich Heterostructures. *Nat. Mater.* **2020**, *19* (7), 732–737. https://doi.org/10.1038/s41563-020-0605-z.

(4) Li, Q.; Trang, C. X.; Wu, W.; Hwang, J.; Cortie, D.; Medhekar, N.; Mo, S.; Yang, S. A.; Edmonds, M. T. Large Magnetic Gap in a Designer Ferromagnet–Topological Insulator–Ferromagnet Heterostructure. *Adv. Mater.* **2022**, *34* (21), 2107520. https://doi.org/10.1002/adma.202107520.

(5) Pereira, V. M.; Wu, C.-N.; Höfer, K.; Choa, A.; Knight, C.-A.; Swanson, J.; Becker, C.; Komarek, A. C.; Rata, A. D.; Rößler, S.; Wirth, S.; Guo, M.; Hong, M.; Kwo, J.; Tjeng, L. H.; Altendorf, S. G. Challenges of Topological Insulator Research: $Bi_2 Te_3$ Thin Films and Magnetic Heterostructures. *Phys. Status Solidi B* **2021**, *258* (1), 2000346. https://doi.org/10.1002/pssb.202000346.

(6) Wang, Y.; Zhang, F.; Zeng, M.; Sun, H.; Hao, Z.; Cai, Y.; Rong, H.; Zhang, C.; Liu, C.; Ma, X.; Wang, L.; Guo, S.; Lin, J.; Liu, Q.; Liu, C.; Chen, C. Intrinsic Magnetic Topological Materials. *Front. Phys.* **2023**, *18* (2), 21304. https://doi.org/10.1007/s11467-022-1250-6.

(7) Tang, Y.-K.; Zhao, W.-J.; Zhu, H.-Q.; Huang, Y.-C.; Cao, W.-W.; Yang, Q.; Yao, X.-Y.; Zhai, Y.; Dong, S. Influence of Vacuum Degree on Growth of $Bi_2 Te_3$ Single Crystal. *Chin. Phys. B* **2015**, *24* (7), 078101. https://doi.org/10.1088/1674-1056/24/7/078101.

(8) Xu, J.-L.; Sun, Y.-J.; He, J.-L.; Wang, Y.; Zhu, Z.-J.; You, Z.-Y.; Li, J.-F.; Chou, M. M. C.; Lee, C.-K.; Tu, C.-Y. Ultrasensitive Nonlinear Absorption Response of Large-Size Topological Insulator and Application in Low-Threshold Bulk Pulsed Lasers. *Sci. Rep.* **2015**, *5* (1), 14856. https://doi.org/10.1038/srep14856.

(9) Zhu, K.; Cheng, Y.; Liao, M.; Chong, S. K.; Zhang, D.; He, K.; Wang, K. L.; Chang, K.; Deng, P. Unveiling the Anomalous Hall Response of the Magnetic Structure Changes in the Epitaxial $MnBi_2 Te_4$ Films. *Nano Lett.* **2024**, *24* (7), 2181–2187. https://doi.org/10.1021/acs.nanolett.3c04095.

(10) Gong, Y.; Guo, J.; Li, J.; Zhu, K.; Liao, M.; Liu, X.; Zhang, Q.; Gu, L.; Tang, L.; Feng, X.; Zhang, D.; Li, W.; Song, C.; Wang, L.; Yu, P.; Chen, X.; Wang, Y.; Yao, H.; Duan, W.; Xu, Y.; Zhang, S.-C.; Ma, X.; Xue, Q.-K.; He, K. Experimental Realization of an Intrinsic Magnetic

Topological Insulator*. *Chin. Phys. Lett.* **2019**, *36* (7), 076801. https://doi.org/10.1088/0256-307X/36/7/076801.

(11) Guo, H.; Bai, C.; Zhu, K.; Lv, S.; Zhai, Z.; Qu, J.; Xian, G.; Han, Y.; Hu, G.; Qi, Q.; Liu, G.; Jiao, F.; Bao, L.; Bao, X.; Liu, X.; Chen, H.; Lin, X.; Zhou, W.; Zhou, J.; Yang, H.; Gao, H.-J. Controllable Synthesis of High-Quality Magnetic Topological Insulator $MnBi_2$ Te_4 and $MnBi_4$ Te_7 Multilayers by Chemical Vapor Deposition. *Nano Lett.* **2024**, *24* (49), 15788–15795. https://doi.org/10.1021/acs.nanolett.4c04700.

(12) Su, S.-H.; Chang, J.-T.; Chuang, P.-Y.; Tsai, M.-C.; Peng, Y.-W.; Lee, M. K.; Cheng, C.-M.; Huang, J.-C. A. Epitaxial Growth and Structural Characterizations of MnBi2Te4 Thin Films in Nanoscale. *Nanomaterials* **2021**, *11* (12), 3322. https://doi.org/10.3390/nano11123322.

(13) Ghasemi, A.; Kepaptsoglou, D.; Figueroa, A. I.; Naydenov, G. A.; Hasnip, P. J.; Probert, M. I. J.; Ramasse, Q.; Van Der Laan, G.; Hesjedal, T.; Lazarov, V. K. Experimental and Density Functional Study of Mn Doped Bi2Te3 Topological Insulator. *APL Mater.* **2016**, *4* (12), 126103. https://doi.org/10.1063/1.4971354.

(14) Mei, R.; Zhao, Y.-F.; Wang, C.; Ren, Y.; Xiao, D.; Chang, C.-Z.; Liu, C.-X. Electrically Controlled Anomalous Hall Effect and Orbital Magnetization in Topological Magnet MnBi 2 Te 4. *Phys. Rev. Lett.* **2024**, *132* (6), 066604. https://doi.org/10.1103/PhysRevLett.132.066604.

(15) Anderson, P. W. New Approach to the Theory of Superexchange Interactions. *Phys. Rev.* **1959**, *115* (1), 2–13. https://doi.org/10.1103/PhysRev.115.2.

(16) Goodenough, J. B. Theory of the Role of Covalence in the Perovskite-Type Manganites [La, M(II)] MnO3. *Phys. Rev.* **1955**, *100* (2), 564–573. https://doi.org/10.1103/PhysRev.100.564.

(17) Kanamori, J. Superexchange Interaction and Symmetry Properties of Electron Orbitals. *J. Phys. Chem. Solids* **1959**, *10* (2–3), 87–98. https://doi.org/10.1016/0022-3697(59)90061-7.

(18) Otrokov, M. M.; Rusinov, I. P.; Blanco-Rey, M.; Hoffmann, M.; Vyazovskaya, A. Yu.; Eremeev, S. V.; Ernst, A.; Echenique, P. M.; Arnau, A.; Chulkov, E. V. Unique Thickness-Dependent Properties of the van Der Waals Interlayer Antiferromagnet MnBi 2 Te 4 Films. *Phys. Rev. Lett.* **2019**, *122* (10), 107202. https://doi.org/10.1103/PhysRevLett.122.107202.

(19) Bhattacharyya, S.; Akhgar, G.; Gebert, M.; Karel, J.; Edmonds, M. T.; Fuhrer, M. S. Recent Progress in Proximity Coupling of Magnetism to Topological Insulators. *Adv. Mater.* **2021**, *33* (33), 2007795. https://doi.org/10.1002/adma.202007795.

(20) Li, B.; Pajerowski, D. M.; Yan, J.-Q.; McQueeney, R. J. Role of Nonmagnetic Spacers in the Magnetic Interactions of Antiferromagnetic Topological Insulators MnBi 4 Te 7 and MnBi 2 Te 4. *Phys. Rev. B* **2025**, *111* (6), 064418. https://doi.org/10.1103/PhysRevB.111.064418.

(21) Song, T.; Fei, Z.; Yankowitz, M.; Lin, Z.; Jiang, Q.; Hwangbo, K.; Zhang, Q.; Sun, B.; Taniguchi, T.; Watanabe, K.; McGuire, M. A.; Graf, D.; Cao, T.; Chu, J.-H.; Cobden, D. H.; Dean, C. R.; Xiao, D.; Xu, X. Switching 2D Magnetic States via Pressure Tuning of Layer Stacking. *Nat. Mater.* **2019**, *18* (12), 1298–1302. https://doi.org/10.1038/s41563-019-0505-2.

(22) Guo, J.; Wang, H.; Zhang, H.; Mi, S.; Li, S.; Dong, H.; Zhu, S.; Hu, J.; Wang, X.; Li, Y.; Sugawara, Y.; Xu, R.; Pang, F.; Ji, W.; Xia, T.; Cheng, Z. Interlayer Coupling Modulated Tunable Magnetic States in Superlattice MnBi 2 Te 4 (Bi 2 Te 3) n Topological Insulators. *Phys. Rev. B* **2024**, *109* (16), 165410. https://doi.org/10.1103/PhysRevB.109.165410.

(23) Trodahl, H. J.; Natali, F.; Ruck, B. J.; Lambrecht, W. R. L. Carrier-Controlled Anomalous Hall Effect in an Intrinsic Ferromagnetic Semiconductor. *Phys. Rev. B* **2017**, *96* (11), 115309. https://doi.org/10.1103/PhysRevB.96.115309.
